# Research progress of connexin 43 in cardiovascular diseases

**DOI:** 10.3389/fcvm.2025.1650548

**Published:** 2025-08-22

**Authors:** Zhao Xinxin, Han Pan, Li Qiao

**Affiliations:** Department of Ultrasonic Medicine, Provincial Hospital Affiliated to Shandong First Medical University, Jinan, Shandong, China

**Keywords:** connexin 43, gap junctions, arrhythmias, myocardial fibrosis, ischemia-reperfusion injury, Chagas disease, mitochondrial function

## Abstract

Gap junctions (GJs) are critical structures for cardiac electrical signal conduction and synchronized contraction. Their fundamental components are transmembrane proteins from the connexin (Cx) family, which assemble into hexameric channels to form intercellular ion-permeable pathways, ensuring efficient electrical transmission and coordinated contraction between cardiac cells. Connexin 43 (Cx43), the most abundant connexin in the heart, serves as the primary constituent of ventricular gap junctions. Alterations in the structure, expression, distribution, and phosphorylation levels of Cx43 are closely associated with various cardiac pathologies, including arrhythmias, myocardial infarction, heart failure, ischemic cardiomyopathy, and diabetic cardiomyopathy. Thus, in-depth investigations into the biological characteristics of Cx43 are essential for elucidating the mechanisms underlying these diseases and developing potential therapeutic strategies. This review summarizes the role of Cx43 in cardiac diseases, explores its functional changes under electrophysiological and pathological conditions, and evaluates its impact on disease progression, providing theoretical insights for mechanistic studies and clinical interventions in cardiovascular diseases.

## Highlights

• Connexin 43 (Cx43) is the primary gap junction protein in cardiac myocytes, critical for electrical conduction and intercellular communication.
• Alterations in Cx43 structure, expression, and phosphorylation are linked to arrhythmias, myocardial infarction, and heart failure.
• Cx43-mediated signaling pathways (Hippo, TGF-β) regulate cardiac fibrosis and ischemic injury.
• Targeting Cx43 phosphorylation and distribution may offer novel therapeutic strategies for cardiovascular diseases.

## 1 Methods

### 1.1 Literature search strategy

The literature search for this review was primarily conducted using internationally recognized academic databases, including PubMed, Web of Science, Embase, and Scopus. The search timeframe spanned from the inception of each database to 2025. The combinations of search keywords included “connexin 43”, “gap junction”, “hemichannel”, “arrhythmia”, etc. Additionally, Medical Subject Headings (MeSH) were used for expanded retrieval to ensure comprehensive coverage of relevant research fields.

### 1.2 Inclusion and exclusion criteria

Inclusion criteria: (1) Published in English; (2) Research content focusing on the molecular structure of Cx43, the functions of gap junctions/hemichannels, and their mechanisms of action in cardiovascular diseases (including Chagas disease); (3) Types of literature, including original research (basic experiments, clinical observations), systematic reviews, and meta-analyses; (4) with complete experimental design or data support.

Exclusion criteria: (1) Duplicated publications; (2) Unpublished materials such as abstracts and conference papers; (3) Research content not directly related to the mechanisms of Cx43 in heart diseases; (4) Literature with obvious methodological flaws or questionable conclusions.

## 2 Structure, distribution, and functions of Cx43

### 2.1 Structure

Cx43 is the principal gap junction protein in mammalian cardiomyocytes, encoded by the connexin alpha 1 gene and composed of 382 amino acids (1).

Gap junctions are intercellular junctional structures composed of clusters of transmembrane channels, whose core function is to mediate the direct cytoplasmic exchange of ions and small-molecule metabolites between adjacent cells (2). The formation of these structures relies on the precise docking and juxtaposition of hemichannels (connexons) expressed by neighboring cells (3). Each hemichannel, functioning as the functional unit of a connexon, pairs with the corresponding hemichannel in an adjacent cell under physiological conditions, collectively forming a channel structure that spans the intercellular space (4). Notably, under specific circumstances such as pathological states or paracrine signaling regulation, individual hemichannels that traverse the entire depth of the plasma membrane can also function as transmembrane channels in non-contacting cells, mediating the transmembrane transport of ions and small-molecule metabolites even in the absence of intercellular contact (5).

**Figure 1:**
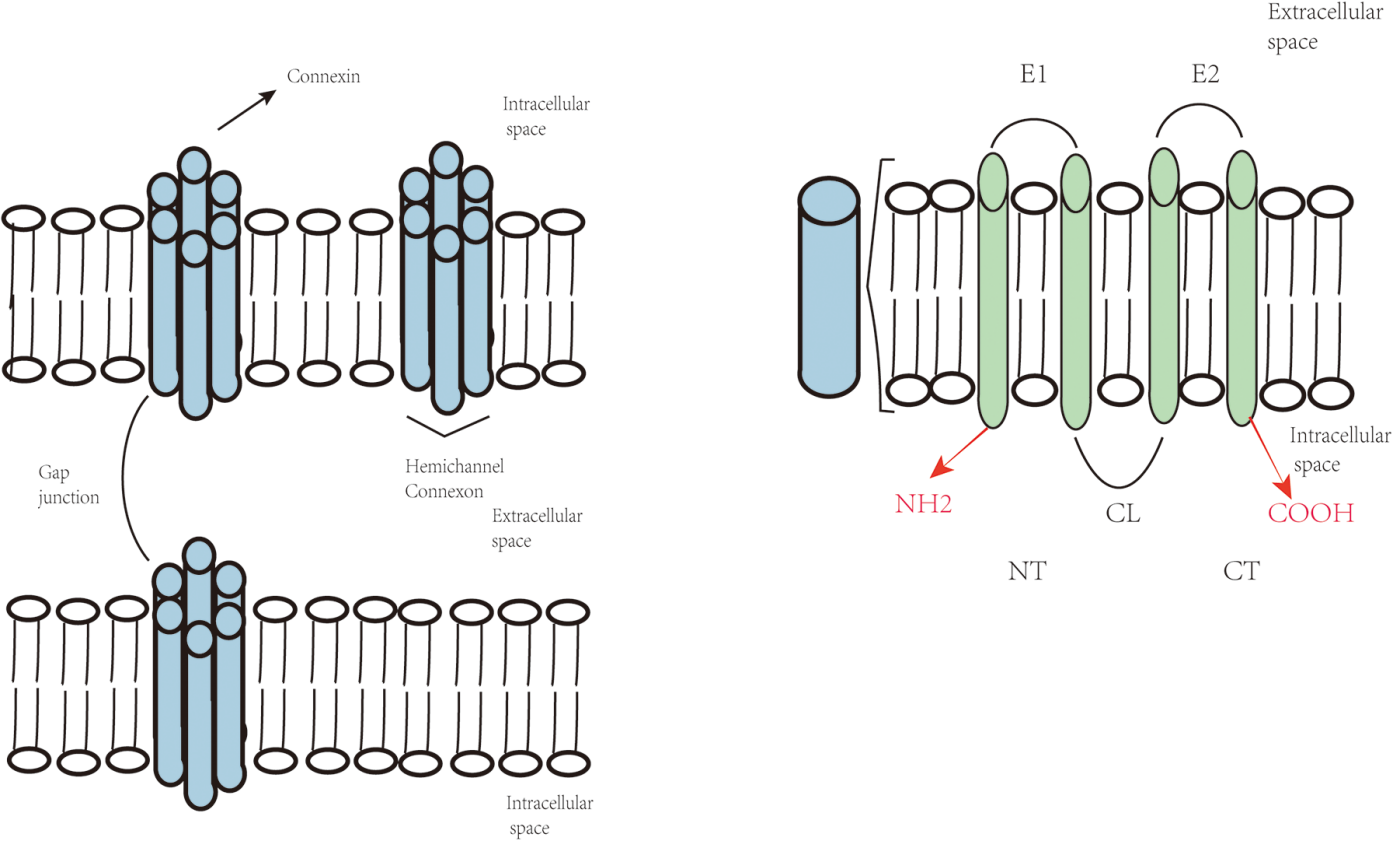
Topological structure of connexins and composition of gap junctions and hemichannels.

Connexins are tetraspan transmembrane domain proteins, characterized by four highly conserved transmembrane domains (M1-M4), intracellular N-terminal and C-terminal regions (NT and CT) (6), which are interconnected by two extracellular loops (E1 and E2) and one cytoplasmic loop (CL) (2). The four transmembrane domains, along with extracellular and intracellular domains, oligomerize into hexameric connexons, which further assemble to form intercellular gap junction channels (2) (Figure 1).

### 2.2 Distribution

Cx43 is widely distributed in the cardiovascular system, predominantly expressed in atrial and ventricular myocytes and the subendocardial conduction system, with high expression in the intercalated discs of ventricular myocytes to support rapid cardiac impulse conduction (7). Cx43 distribution in cardiomyocytes is tissue-specific, primarily localized at the end-to-end connections of intercalated discs (8). This distribution is crucial for maintaining electrical and mechanical coupling between cardiomyocytes (9). Studies indicate that in addition to forming classical gap junctions in intercalated discs, Cx43 can be localized to the inner and outer mitochondrial membranes via a mitochondrial targeting sequence (MTS) in its amino terminus (10). It forms complexes with voltage-dependent anion channels (VDAC) to regulate mitochondrial permeability transition pore (mPTP) opening and mediate mitochondrial dynamics and oxidative stress responses (11). Abnormal mitochondrial Cx43 is closely associated with heart failure, acute myocardial infarction, ischemia-reperfusion injury, arrhythmias, diabetic cardiomyopathy, and hypertensive heart disease (12).

### 2.3 Differential roles of Cx43 gap junctions and hemichannels

Cx43 can form classical gap junctions (GJs) between adjacent cardiomyocytes or exist independently as hemichannels (HCs) on the plasma membrane (13). These two forms exhibit distinct physiological and pathological functions and contribute differentially to cardiac disease mechanisms (13).

#### 2.3.1 Cx43 gap junctions

Cx43-based gap junctions are composed of two hemichannels contributed by adjacent cells, enabling the direct intercellular transfer of ions and small metabolites (14). This electrical and metabolic coupling is essential for synchronized myocardial contraction and normal cardiac rhythm. Disruption in Cx43 expression, localization, or phosphorylation impairs gap junction integrity, leading to electrical conduction abnormalities and an increased risk of arrhythmias (14).

#### 2.3.2 Cx43 hemichannels

In contrast, Cx43 hemichannels remain predominantly closed under physiological conditions but can aberrantly open in response to pathological stimuli such as ischemia, oxidative stress, or inflammation (13). This aberrant activation results in the extracellular release of ATP, glutamate, reactive oxygen species (ROS), and the influx of calcium ions, exacerbating cellular injury and pro-inflammatory signaling (14). Studies have implicated dysfunctional Cx43 HCs in the pathogenesis of myocardial ischemia-reperfusion injury, myocarditis, and heart failure (14).

As summarized in Table 1, Cx43 GJs and HCs differ significantly in structure, function, and disease involvement. Cx43 GJs primarily maintain cardiac electrical synchrony, with their dysfunction contributing to arrhythmogenesis (14). Conversely, pathological opening of Cx43 HCs acts as a critical driver of cardiomyocyte injury, apoptosis, and inflammatory propagation (15). Therefore, selective modulation of GJ and HC activity, such as enhancing gap junction communication or inhibiting hemichannel opening, represents a promising therapeutic strategy for various cardiac pathologies (15).

**Table 1:** Differences between Cx43 gap junctions and hemichannels in cardiac function and disease.

Feature	Cx43 gap junctions (GJs)	Cx43 hemichannels (HCs)
Structure	Formed by two hemichannels from adjacent cells	Single hemichannel on the plasma membrane
Physiological State	Normally open to allow intercellular communication	Normally closed under physiological conditions
Function	Enables electrical and metabolic coupling between cardiomyocytes	Releases signaling molecules; permits Ca^2+^ influx under stress
Regulation	Controlled by phosphorylation and membrane localization	Activated by ischemia, oxidative stress, inflammation
Pathological Role	Impairment leads to conduction defects and arrhythmias	Aberrant opening promotes cell injury, apoptosis, and inflammation
Associated Conditions	Arrhythmias, conduction disorders	Ischemia-reperfusion injury, myocarditis, heart failure
Therapeutic Targeting	Enhancers of GJ function to restore conduction	Specific HC blockers to reduce cell damage and inflammation

## 3 Cx43 and arrhythmias

Cx43 is the primary structural component of gap junctions in cardiomyocytes. It can assemble into classical gap junctions (GJs) that enable direct intercellular transmission of electrical signals and small molecules, or exist as hemichannels (HCs) on the plasma membrane of individual cells, mediating pathological signal release and ion flux (14). These two distinct forms of Cx43 play complementary yet divergent roles in the initiation and maintenance of cardiac arrhythmias.

### 3.1 Cx43 gap junction dysfunction

Growing evidence indicates that Cx43 gap junctions (GJs) play a critical role in arrhythmogenesis by contributing to electrical uncoupling and structural remodeling (13). Cx43 GJs ensure the rapid and coordinated propagation of electrical impulses between cardiomyocytes (14). However, reduced expression, aberrant phosphorylation, or mislocalization of Cx43 impairs gap junctional conduction, resulting in slowed impulse propagation and increased electrical heterogeneity—key substrates for reentrant arrhythmias (14).

In pulmonary hypertension-associated arrhythmias, downregulation and disorganization of Cx43 have been shown to significantly increase arrhythmic susceptibility (15). In diabetic cardiomyopathy, Cx43 expression is reduced and its distribution altered, predisposing the heart to ventricular arrhythmias (15). During early cardiac hypertrophy, transient upregulation of Cx43 may serve to preserve electrical stability, but this is followed by a decline in expression and redistribution, characterized by decreased localization at the intercalated disks and increased lateral membrane presence—changes associated with elevated arrhythmia risk (16). Similarly, ischemic conditions induce Cx43 downregulation and lateralization from intercalated disks to transverse sarcolemmal regions, collectively contributing to arrhythmic events (16).

In rabbit models of myocardial infarction, Cx43 expression is markedly reduced in both infarcted and peri-infarct regions, with a positive correlation between the extent of downregulation and the incidence of arrhythmias (16), underscoring Cx43's essential role in maintaining cardiac conduction.

Furthermore, the functional state of Cx43 is closely regulated by its phosphorylation status (17). Under pathological conditions such as myocardial ischemia, oxidative stress, or hyperglycemia, excessive activation of protein kinase C (PKC) leads to abnormal phosphorylation at the serine 368 (S368) site of Cx43, which has been implicated in arrhythmogenesis (16).

Notably, studies on coronary artery disease–related arrhythmias have shown that pharmacological Cx43 enhancers can improve intercellular electrical conduction and reduce the occurrence of arrhythmias, thereby improving patient outcomes (17). Collectively, these findings suggest that targeted modulation of Cx43 expression, distribution, and phosphorylation may offer novel therapeutic strategies for the prevention and management of cardiac arrhythmias (17).

### 3.2 Aberrant opening of Cx43 hemichannels and its role in cardiac arrhythmias

The pathological opening of Cx43 hemichannels (HCs) has been implicated in multiple mechanisms contributing to the initiation and maintenance of cardiac arrhythmias (14).

Firstly, under pathological stimuli, abnormal HC opening facilitates excessive release of intracellular ATP (15). In disease conditions, extracellular ATP accumulation can activate purinergic receptors such as P2X7, leading to intracellular calcium overload and sodium influx. These ionic disturbances promote delayed afterdepolarizations (DADs) and triggered activity, which are recognized as key initiating events in arrhythmogenesis (15).

Secondly, calcium influx through open hemichannels disrupts intracellular calcium homeostasis, directly altering the electrophysiological properties of cardiomyocytes (15). Calcium overload not only enhances sarcoplasmic reticulum (SR) calcium leak but also activates calcium-dependent enzymes such as calpains and phospholipases, leading to compromised membrane stability and abnormal action potential generation, thereby increasing arrhythmia susceptibility (16).

In addition, the release of glutamate and reactive oxygen species (ROS) via Cx43 HCs exacerbates local oxidative stress and excitotoxicity, further destabilizing membrane potential and promoting ectopic pacemaker activity and reentry-based arrhythmias (16). These microenvironmental alterations can also exert paracrine effects on neighboring cells, expanding the arrhythmogenic substrate (16).

**Figure 2:**
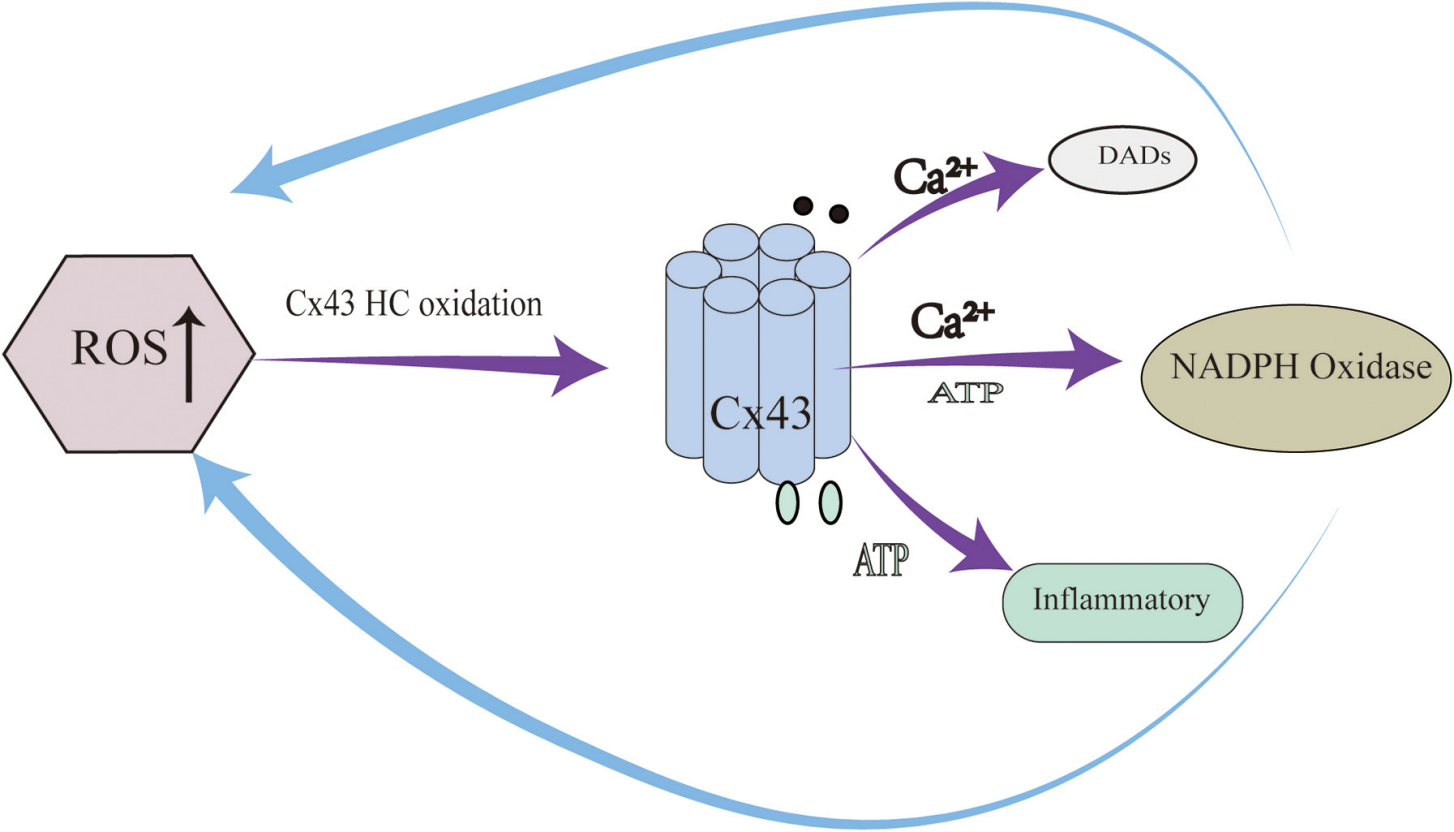
ROS–hemichannel positive feedback loop plays a critical role in arrhythmogenesis.

Notably, Cx43 hemichannels participate in a self-amplifying “ROS–hemichannel positive feedback loop” that plays a critical role in arrhythmogenesis (Figure 2). Under oxidative stress, elevated ROS levels oxidize Cx43 HCs, promoting their pathological opening and resulting in further ATP and Ca^2+^ release. This, in turn, activates NADPH oxidase in adjacent cells, leading to enhanced ROS production and a vicious cycle of oxidative damage and hemichannel activation (17). This feedback loop promotes arrhythmias through three parallel mechanisms: (1) ROS-induced Cx43 internalization and degradation, causing slowed conduction and electrical heterogeneity; (2) Ca^2+^ overload–mediated DADs via hemichannel activity; and (3) ATP-induced inflammatory signaling. Importantly, this loop exhibits a spatial amplification effect, whereby hemichannel activation in a single cell may induce responses in 5–8 neighboring cells (16).

Furthermore, Cx43 HC opening is often accompanied by a decline in gap junction function (14). Excessive HC activation can promote Cx43 internalization and degradation, reducing the number of functional gap junctions and weakening electrical coupling, thereby facilitating conduction block and reentry (15).

Although these findings highlight the pivotal role of Cx43 hemichannels in arrhythmia pathogenesis, current anti-arrhythmic therapies do not target hemichannel activity (16). Future research should aim to elucidate the spatiotemporal characteristics of Cx43 HC activation and downstream signaling cascades, and to develop selective HC inhibitors that preserve gap junction function, offering new prospects for precise and effective arrhythmia management (17).

Atrial fibrillation (AF), the most common rapid atrial arrhythmia, is characterized by significantly reduced Cx43 expression in atrial myocardium (15). In angiotensin II-induced murine AF models, decreased Cx43 protein levels coincide with S368 dephosphorylation and lateral redistribution from intercalated discs (18). Yang et al. demonstrated that Cx43 overexpression reduces obstructive sleep apnea (OSA)-associated AF via the CaMKⅡγ/HIF-1 signaling axis (19).

The core mechanism of AF aligns with the “AF begets AF” theory, wherein AF drives atrial electrical and structural remodeling. Cx43 plays a central role in these remodeling processes (2). Interactions between Cx43 and multiple proteins/ion channels contribute to AF-related electrical remodeling (2). Oxidative stress, a key driver of AF progression, induces Cx43 phosphorylation and degradation via elevated reactive oxygen species (ROS), exacerbating electrical remodeling and AF susceptibility (2). Weakened interactions between Cx43 and calcium channels further impair calcium handling, aggravating AF pathology (2).

AF-induced structural remodeling involves chronic pathological changes, including extracellular matrix remodeling, myocardial fibrosis, and cellular phenotypic switching (19). Cx43-regulated fibrotic processes are central to this remodeling, marked by increased collagen volume fraction, elevated myofibroblast density, mitochondrial fragmentation, and intercalated disc disruption (20).

## 4 Cx43 and Chagas disease

### 4.1 Pathological features of Chagas disease and physiological basis of gap junctions

Chagas disease, caused by infection with the protozoan parasite Trypanosoma cruzi, is a neglected tropical disease with distinct clinical phases: acute, indeterminate, and chronic (21). Approximately 30% of chronically infected individuals progress to chronic Chagas cardiomyopathy (CCC), characterized by life-threatening cardiac manifestations including conduction abnormalities, ventricular arrhythmias, cardiomegaly, and heart failure (22).

Mounting evidence indicates that dysregulation of Cx43—manifested by altered expression, aberrant localization, and structural remodeling of gap junctions—constitutes a pivotal mechanism underlying Chagas disease-associated myocardial injury (23). These Cx43 abnormalities are closely associated with impaired electrical coupling and contractile dysfunction in the infected heart (24).

### 4.2 Abnormalities and mechanisms of connexin 43 in Chagas disease

#### 4.2.1 Alterations in Cx43 expression and distribution

In the acute phase of infection: During the initial stage of Trypanosoma cruzi infection, the expression level of Cx43 in cardiomyocytes transiently increases, which may be associated with elevated phosphorylation of Cx43 (25). One hour post-infection, the phosphorylated forms of Cx43 (P1 + P2) show a more significant increase compared to the non-phosphorylated form (P0), suggesting that Cx43 phosphorylation might be involved in the early infection process (25).

In the chronic phase of infection: With the persistence of infection, Cx43 expression gradually decreases, showing a significant reduction in the late stage of infection (26). Chronic infection leads to a decrease in total Cx43 content, disrupts the normal distribution pattern of Cx43, and causes Cx43 to disperse from the intercalated disc region to the lateral cell membrane and cytoplasm (27). Table 2 delineates stage-specific Cx43 perturbations in T. cruzi infection, emphasizing its role as a critical bridge between pathogen invasion and end-stage cardiac damage.

**Table 2:** Condensed summary of Cx43 abnormalities in T. *cruzi* infection.

Stage	Cx43 abnormalities	Mechanisms	Consequences
Acute	1. Reduced expression 2. Ectopic localization (intercalated discs → cytoplasm/lateral membranes)	*T. cruzi* direct interference with Cx43 synthesis/trafficking	↓ Electrical coupling, conduction slowing, fatal arrhythmias
Chronic	1. Dysfunctional channel assembly 2. ↓ Channel density 3. Hemichannel imbalance	Cytokines (TNF-α, IL-1β) activate JNK/PKC; Cx43 S368 phosphorylation	Intercellular communication uncoupling, dilated cardiomyopathy, conduction block

#### 4.2.2 Abnormal Cx43 phosphorylation

Trypanosoma cruzi infection can alter the phosphorylation status of Cx43 (27). In the acute phase, the phosphorylated forms of Cx43 increase, which may be related to factors such as elevated intracellular calcium concentration and activation of inflammatory cytokines (25). In the chronic phase, the phosphorylation pattern of Cx43 changes; for instance, the phosphorylation levels and distribution of sites such as Cx43 S368 and Cx43 S325/328/330 become abnormal (27).

#### 4.2.3 Role of inflammatory cytokines

Trypanosoma cruzi infection triggers a robust myocardial inflammatory response, leading to excessive production of inflammatory cytokines such as TNF-α, IFN-γ, and IL-1β. These cytokines can affect the expression and function of Cx43 through multiple pathways (28). For example, TNF-α and IFN-γ can induce downregulation of Cx43 expression and alter its distribution; inflammatory cytokines can also activate signaling pathways such as JAK/STAT, further influencing the phosphorylation status and intracellular localization of Cx43 (29).

#### 4.2.4 Oxidative stress and metabolic disorders

Trypanosoma cruzi infection induces metabolic reprogramming in cardiomyocytes, characterized by enhanced oxidative phosphorylation and uncoupling of ATP production from oxygen consumption, resulting in massive generation of reactive oxygen species (ROS) (29). ROS can oxidize Cx43, causing abnormalities in its structure and function, such as pathological opening of hemichannels (26). Additionally, metabolic disorders can affect the synthesis, degradation, and transport of Cx43, further impairing its normal function (27).

#### 4.2.5 Impaired gap junction function

Abnormalities in Cx43 expression, distribution, and phosphorylation ultimately lead to impaired gap junction function (27). The number of gap junctions decreases, conduction velocity slows down, and electrical coupling between cells weakens, thereby increasing the risk of arrhythmias (25). In the chronic phase of infection, the lateral distribution and dephosphorylation of Cx43 can cause gap junction conduction block, forming a substrate for arrhythmias (28).

#### 4.2.6 Occurrence of arrhythmias

Cx43 abnormalities induced by Trypanosoma cruzi infection can alter the electrophysiological properties of cardiomyocytes, such as shortening of action potential duration, conduction block, and delayed afterdepolarization, thereby triggering various arrhythmias (29). Cx43 abnormalities can also affect calcium homeostasis in cardiomyocytes, further promoting the occurrence of arrhythmias (30).

In summary, Trypanosoma cruzi infection induces Cx43 abnormalities in cardiomyocytes through multiple mechanisms, including alterations in Cx43 expression and distribution, abnormal phosphorylation, effects of inflammatory cytokines, oxidative stress, and metabolic disorders (28). These mechanisms collectively contribute to impaired gap junction function and arrhythmias, and they are interconnected in the pathological process of myocardial lesions caused by Trypanosoma cruzi infection (30). Table 3 provides a concise summary of the mechanisms underlying Trypanosoma cruzi–induced Cx43 abnormalities in cardiomyocytes, including specific alterations, stages of infection, and functional consequences.

**Table 3:** Mechanisms of trypanosoma cruzi–induced Cx43 abnormalities in cardiomyocytes.

Mechanistic category	Specific alterations	Stage of infection	Functional consequences
Cx43 Expression and Redistribution	Acute: transient upregulation; chronic: downregulation and lateralization.	Acute chronic	Disrupted gap-junction formation and function.
Cx43 Phosphorylation	Acute: increased phosphorylation; chronic: altered phosphorylation sites.	Acute chronic	Altered Cx43 conformation and stability.
Inflammatory Cytokines	Elevated TNF-α, IFN-γ, IL-1β; downregulation and redistribution of Cx43	Chronic	Amplified inflammation and Cx43 loss
Oxidative Stress & Metabolic Dysregulation	ROS-induced Cx43 oxidation; impaired synthesis and trafficking	Chronic	Pathological hemichannel activity and dysfunction.
Gap-Junction Dysfunction	Reduced plaque number, slower conduction, weakened coupling.	Chronic	Arrhythmogenic substrate
Arrhythmia Development	Altered electrophysiology: shortened action potentials, conduction block, DADs	Chronic	Onset of arrhythmias and impaired cardiac function

### 4.3 Association with fibrosis and heart failure

Chronic Cx43 deficiency contributes to myocardial interstitial fibrosis, likely by promoting fibroblast activation and excessive collagen deposition (25). Moreover, impaired gap junction communication diminishes synchronous contraction, increases mechanical stress, and promotes ventricular remodeling, ultimately leading to heart failure (26).

### 4.4 Clinical implications and targeted therapeutic strategies

Abnormalities in Cx43 represent the core mechanism underlying arrhythmias in patients with chronic Chagas disease (31). These abnormalities disrupt the synchrony of myocardial electrical conduction, triggering reentrant arrhythmias and other rhythm disturbances, which constitute one of the leading causes of mortality in affected individuals (30).

In terms of therapeutic exploration, de Oliveira et al. (31) demonstrated that the TGF-β inhibitor GW788388 improves cardiac conduction by suppressing fibrosis and restoring the proper localization of Cx43. Additionally, Cx43 openers and blockers have shown potential in regulating electrophysiological function in experimental models; however, their clinical translation requires further validation (31).

## 5 Cx43 and myocardial fibrosis

Cardiac fibrosis is a pathological process characterized by the abnormal proliferation of fibrous connective tissue in the heart, often accompanied by structural changes and impaired cardiac function (32). In recent years, the role of Cx43 in the development and progression of cardiac fibrosis has garnered significant attention. Cx43 dephosphorylation is considered a predisposing factor for cardiac fibrosis, affecting the survival and function of cardiomyocytes (33). Studies have shown that Cx43 dephosphorylation can induce cardiomyocyte apoptosis, thereby promoting fibrotic progression (34). Consequently, the phosphorylation state of Cx43 has emerged as a potential therapeutic target for anti-fibrotic interventions (33).

**Figure 3:**
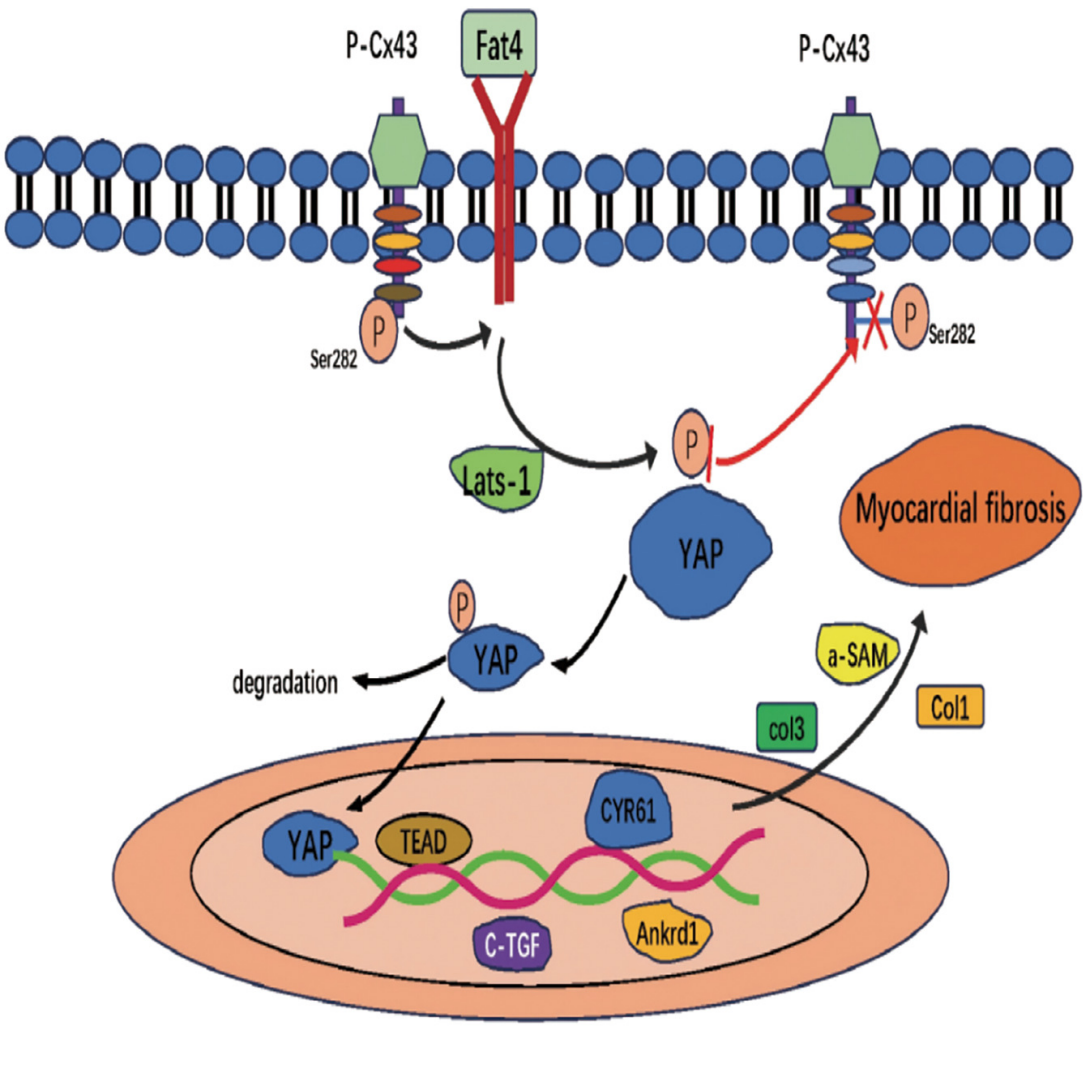
Hippo signaling pathway promotes cardiac fibrosis.

Hyperphosphorylation of Cx43 at the S282 site has been linked to cardiomyocyte apoptosis and cardiac fibrosis (35). Both *in vitro* and *in vivo* experiments demonstrate that imbalances in Cx43 S282 phosphorylation lead to cardiomyocyte dysfunction and fibrotic progression (35). Cx43 S282 phosphorylation influences fibrosis through the Hippo signaling pathway (36). Research indicates that defective Cx43 S282 phosphorylation downregulates Dchs1 gene expression, leading to inhibition of YAP phosphorylation. Unphosphorylated YAP translocates to the nucleus, binds to TEAD, and activates target gene transcription, thereby promoting fibrotic progression (36) (Figure 3). Thus, Cx43 S282 phosphorylation upregulates Dchs1 expression, inhibits YAP dephosphorylation and nuclear translocation, and suppresses the Hippo signaling pathway, ultimately alleviating cardiac fibrosis (37). This mechanism provides a novel molecular target for the treatment of cardiac fibrosis.

In cardiac fibrosis, Cx43 expression is typically significantly reduced (38). This change is considered an adaptive response in cardiac remodeling and may lead to reduced intercellular electrical signaling between cardiomyocytes, thereby providing a substrate for arrhythmogenesis (38). Reduced Cx43 expression is also associated with increased fibroblast activity. Fibroblasts are key cells responsible for synthesizing and degrading components of the cardiac extracellular matrix (39). Decreased Cx43 expression enhances fibroblast activity, leading to increased collagen deposition and exacerbating the progression of cardiac fibrosis (38). As Cx43 expression declines, collagen deposition in cardiac tissue increases, affecting both the structure and function of the heart. Excessive collagen deposition can cause cardiac stiffness, reduce cardiac compliance, and impair systolic and diastolic function (40).

**Figure 4:**
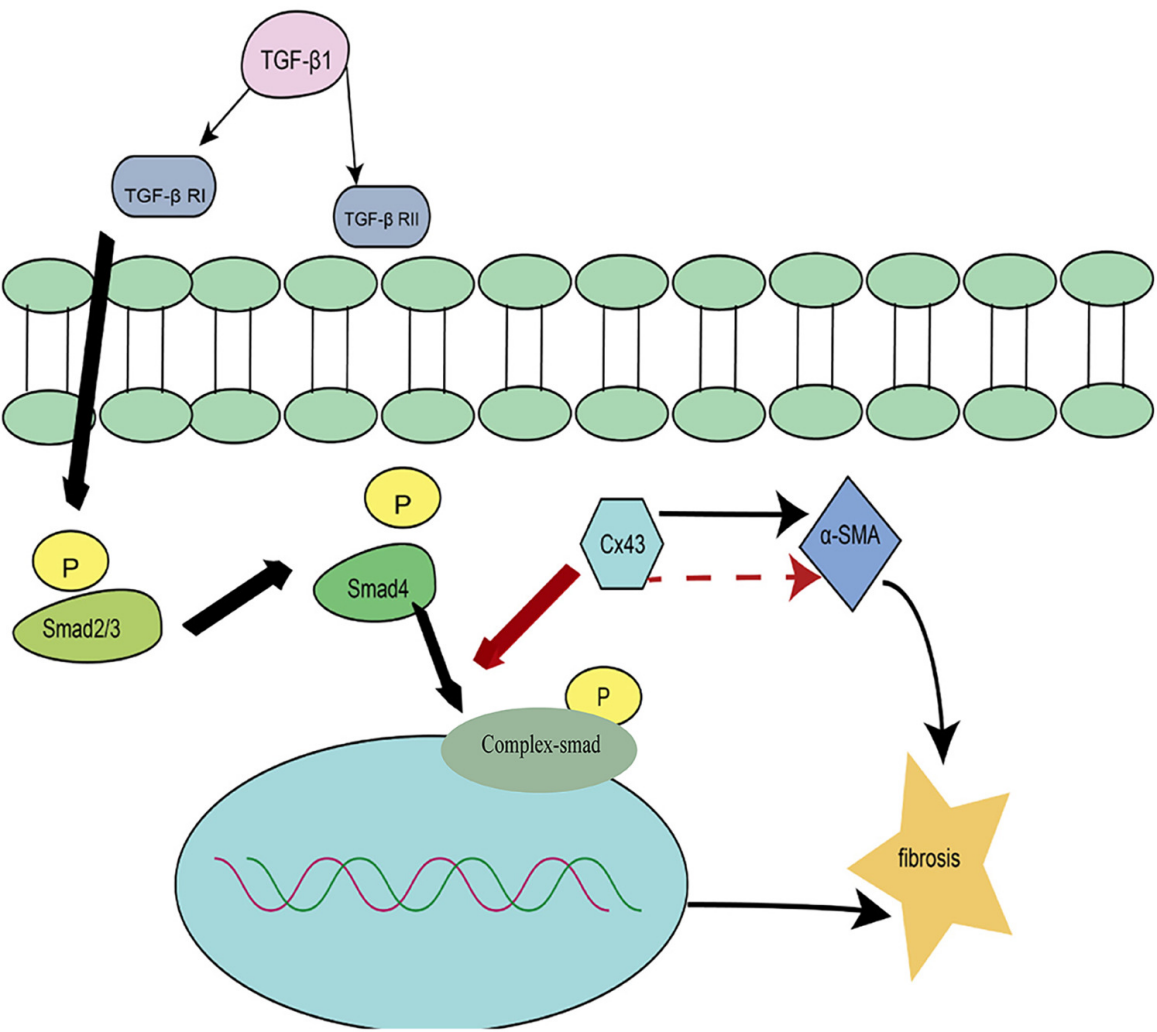
Regulation of cardiac fibrosis by the TGF-β signaling pathway.

Cx43 plays a critical role in transforming growth factor-β (TGF-β) signaling, regulating the differentiation of cardiac fibroblasts into myofibroblasts (41). Studies have found that in cultured neonatal rat cardiac fibroblasts, the expression levels of α-smooth muscle actin (α-SMA) are positively correlated with Cx43. Inhibiting endogenous Cx43 activity significantly downregulates α-SMA expression, whereas Cx43 overexpression promotes upregulation of α-SMA (41) (Figure 4). Experimental evidence suggests that Cx43 mediates TGF-β signaling to regulate α-SMA expression, playing a key role in cardiac fibrosis.

The role of Cx43 in cardiac fibrosis directly impacts the electrophysiological properties and structural remodeling of the heart (42). Future research should focus on exploring ways to modulate Cx43 expression to intervene in cardiac fibrosis, thereby improving cardiac function and patient outcomes. In summary, changes in Cx43 expression are closely associated with cardiac fibrosis, revealing a complex biological mechanism involving intercellular electrical signaling, fibroblast activity, and cardiac structural remodeling. A deeper understanding of these mechanisms will enhance our comprehension of cardiac fibrosis progression and provide new directions for clinical therapy.

## 6 Cx43 and ischemic heart disease

### 6.1 Stable coronary artery disease (SCAD)

Stable coronary artery disease (SCAD) is characterized pathologically by fixed coronary artery stenosis (stenosis degree >50%) (42). The stability of its clinical symptoms depends on the effective establishment of collateral circulation. Recent studies have revealed that connexin 43 plays a protective role in collateral circulation formation in SCAD through both channel-dependent and non-channel-dependent mechanisms (43).

**Figure 5:**
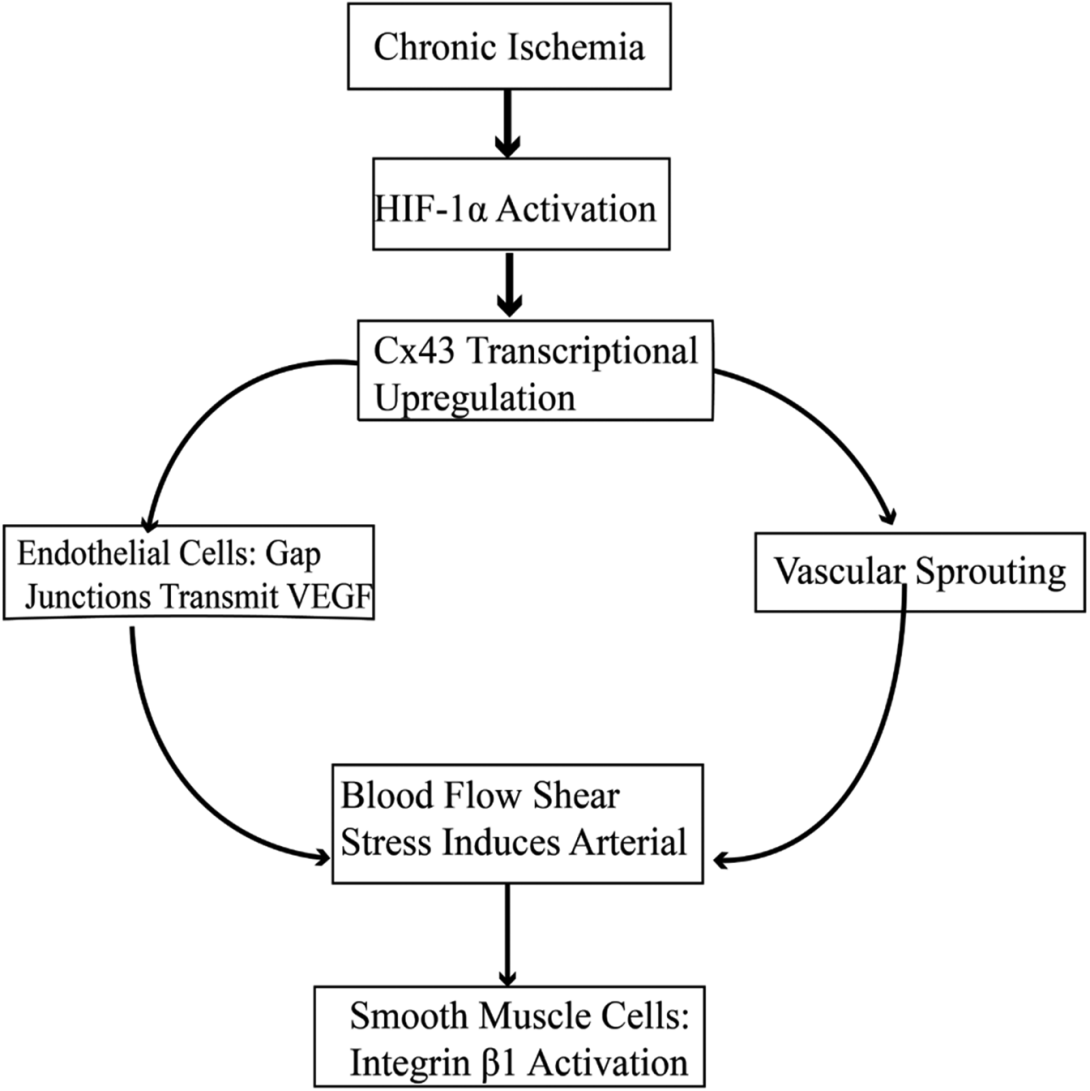
Cx43-Mediated signaling pathways in collateral circulation formation.

In collateral circulation formation in stable angina, Cx43 plays a central role through endothelial cell—smooth muscle cell coordinated regulation. In endothelial cells, ischemic stress induces upregulation of Cx43 expression via the hypoxia-inducible factor 1α (HIF-1α) pathway (42). On the one hand, gap junction channels transmit vascular endothelial growth factor (VEGF-A) to adjacent cells, promoting vascular sprouting; on the other hand, Cx43 hemichannels are activated, releasing ATP to activate P2Y2 receptors in surrounding cells and initiating the ERK1/2 phosphorylation cascade (43). In smooth muscle cells, the carboxyl terminus of Cx43 directly binds to integrin β1, enhancing α—smooth muscle actin (α—SMA) expression and extracellular matrix deposition, thereby expanding the diameter of the neovascular lumen. At the same time, Cx43—mediated Ca^2+^ waves coordinate smooth muscle cell contraction, optimizing blood flow distribution to promote vascular maturation (44). This dual—cell regulatory network together constitutes the molecular basis of Cx43 in collateral circulation formation (40) (Figure 5).

### 6.2 Acute coronary syndrome (ACS)

#### 6.2.1 Cx43 and ST-segment elevation myocardial infarction (STEMI)

Studies have shown that the expression, distribution, and phosphorylation abnormalities of Cx43 are closely related to the occurrence of myocardial infarction (45). Cx43 remodeling is involved in the occurrence and progression of myocardial infarction, and it may exacerbate ischemic injury after myocardial infarction by affecting metabolic coupling of cardiomyocytes (44). In the acute phase of STEMI (0–6 h after onset), ischemia and hypoxia caused by complete coronary artery occlusion can rapidly induce post-translational modifications of Cx43, particularly the phosphorylation level of serine 368 (pS368), which is significantly increased (44). This post-translational modification disrupts the interaction between Cx43 and plaque proteins (such as ZO-1), leading to the disassembly of gap junctions and their internalization from the cell membrane to the cytoplasm, forming the so-called “electrical uncoupling” phenomenon (43).

In the subacute phase (1–7 days), surviving cardiomyocytes around the infarct upregulate Cx43 expression compensatorily. However, due to the continuous stimulation of inflammatory factors (such as TNF-α) in the ischemic microenvironment, Cx43 exhibits disordered clustered distribution, which is prone to inducing arrhythmias (44). In myocardial infarction, the phosphorylation of Cx43 affects the infarct size and prognosis through a triple mechanism of electrical coupling regulation, mitochondrial protection, and oxidative stress (45). Therefore, modulating the expression, distribution, and phosphorylation levels of Cx43 may provide new intervention strategies for the prevention and treatment of myocardial infarction.

#### 6.2.2 Cx43 and non-ST-segment elevation myocardial infarction (NSTEMI) and unstable angina (UA)

In patients with non-ST-segment elevation myocardial infarction (NSTEMI) and unstable angina (UA), myocardial ischemic injury leads to a decrease in Cx43 expression, lateral changes in distribution, and a significant increase in dephosphorylation, which is positively correlated with the severity of myocardial ischemia (46). Myocardial ischemia induces the dephosphorylation of Cx43 through certain mechanisms. Under ischemic conditions, the intracellular oxidative stress level is significantly increased, activating a series of protein phosphatases. These enzymes directly act on the phosphorylation sites of Cx43, promoting its dephosphorylation (46). In addition, ischemia may indirectly regulate the phosphorylation state of Cx43 by affecting intracellular calcium ion homeostasis (46).

Compared with patients with STEMI, the downregulation of Cx43 expression in the myocardium of NSTEMI patients is less severe, but it is accompanied by inflammation factor-mediated inhibition of Cx43 function (47). Cx43 is involved in the pathological process through a triple pathway of platelets, myocardium, and microvasculature (46). Compared with STEMI, its characteristic manifestations are thrombosis dominated by platelet Cx43, inflammatory regulation dominated by S262 phosphorylation, and arrhythmia mechanisms dominated by conduction heterogeneity rather than electrical uncoupling (48). Long-term myocardial ischemia activates Cx43 in myocardial fibroblasts, promoting their proliferation and collagen secretion, thereby leading to myocardial fibrosis (46).

## 7 Cx43 and myocardial ischemia-reperfusion injury and cardioprotection

Myocardial ischemia-reperfusion injury (MIRI) is a common pathological phenomenon during the treatment of patients with coronary heart disease, characterized by aggravated myocardial cell injury after reperfusion (47). Its mechanisms involve oxidative stress, inflammatory responses, cell apoptosis, and electrical remodeling (47).

Studies have shown that in ischemic conditions, the expression and phosphorylation of Cx43 decrease, and gap junctions are impaired, leading to abnormal intercellular communication among cardiomyocytes. A portion of Cx43 is translocated to mitochondria, which may be associated with ischemic tolerance (47). During the reperfusion phase, Cx43 undergoes abnormal degradation and redistribution, with a reduced proportion of phosphorylated Cx43 and an increased proportion of dephosphorylated Cx43 (48).

Research indicates that dephosphorylation of Cx43 at the S282 site is closely related to MIRI, and Cx43 may contribute to MIRI by inducing cardiomyocyte apoptosis and fibrosis through the activation of the p38/Fas/FADD signaling pathway (48). It has also been found that mitochondrial Cx43 plays a significant role during ischemia-reperfusion. Abnormal accumulation of mitochondrial Cx43 in the infarct border zone promotes the opening of the mitochondrial permeability transition pore (mPTP). Meanwhile, hemichannels mediate Fe^2+^ influx, synergistically exacerbating cell death (46). Therefore, regulating the expression and distribution of Cx43 can reduce myocardial injury caused by reperfusion. For instance, certain Cx43 modulators can enhance gap junction function, decrease the apoptosis rate of cardiomyocytes, and improve cardiac function (47).

Cx43 is not only involved in the injury process during MIRI but also plays a cardioprotective role in ischemic preconditioning (IPC) (45). It has been found that IPC can enhance the expression of mitochondrial Cx43, reduce reactive oxygen species (ROS) generation, stabilize mitochondrial membrane potential, and increase the tolerance of cardiomyocytes to ischemia-reperfusion injury, thereby reducing cell apoptosis (44). Cx43 is involved in the regulation of ATP-sensitive potassium channels (mitoKATP), and its activation can alleviate calcium overload and oxidative stress induced by ischemia-reperfusion (45). Moreover, targeting mitochondrial Cx43 can simultaneously inhibit apoptosis and ferroptosis (46). Thus, modulating the structure and function of Cx43 can serve as a therapeutic target for post-ischemia-reperfusion cardioprotection.

## 8 Cx43 and cardiac hypertrophy

Cardiac hypertrophy is a significant stage in the progression of cardiovascular diseases, involving myocardial cell remodeling, interstitial fibrosis, and electrical remodeling (47). Its occurrence and progression are closely related to changes in the expression and function of Cx43 (48).

Cardiac hypertrophy is an adaptive response of the heart to mechanical load or neurohumoral stimuli. However, long-term excessive hypertrophy can lead to the development of heart failure (49). Studies have shown that Cx43 undergoes dynamic changes during the process of cardiac hypertrophy, and its expression levels and localization are crucial for the function of cardiomyocytes (39). Cx43 expression is bidirectionally regulated in different types of cardiac hypertrophy (39). Research indicates that in physiological hypertrophy, the expression level of Cx43 remains stable or even increases, thereby maintaining normal cardiac electrical activity and contractile function (50). However, in pathological cardiac hypertrophy, Cx43 expression is often downregulated, accompanied by abnormal distribution of gap junctions (39). For example, Duffy et al. (41) found in a hypertension mouse model that the expression of Cx43 in gap junctions between cardiomyocytes decreased, leading to a reduction in ventricular conduction velocity and an increased risk of arrhythmias.

Moreover, post-translational modifications of Cx43 play a key role in the process of cardiac hypertrophy (3, 39). Cx43 affects the stability and function of gap junctions through phosphorylation modifications mediated by different kinases. Studies have shown that in the early stages of cardiac hypertrophy, phosphorylation at specific sites may contribute to adaptive responses, while sustained abnormal phosphorylation promotes pathological remodeling (46).

## 9 Cx43 and diabetic cardiomyopathy

Diabetic cardiomyopathy (DCM) is a condition characterized by myocardial structural and functional abnormalities caused by diabetes, independent of coronary artery disease and hypertension. Its features include myocardial hypertrophy, interstitial fibrosis, contractile and relaxant dysfunction, and arrhythmias (41). Cx43 plays a significant role in the progression of DCM. In DCM models, the gene and protein expression levels of Cx43 are significantly reduced, and its distribution is disrupted, affecting cardiac function (46). Cx43 may be involved in insulin resistance by regulating the Akt/AMPK signaling pathway (43). Its abnormal expression may exacerbate myocardial glucose and fatty acid utilization disorders, further impairing cardiac function (42).

Cx43 interacts with phosphatidylethanolamine-binding protein 1, mediating ferroptosis and triggering diabetic myocardial ischemia-reperfusion injury (39). Mitochondrial Cx43 is crucial for maintaining myocardial energy metabolism and antioxidant defense. In diabetic states, reduced mitochondrial Cx43 expression makes the myocardium more susceptible to oxidative stress damage, lowers mitochondrial ATP production, and affects cardiac contractile function (41). Targeting Cx43 may offer new therapeutic directions for preventing and treating DCM, providing a theoretical basis for understanding cardiac disease mechanisms and progression, though further research is needed to clarify specific mechanisms.

## 10 Cx43 and heart failure

Heart failure (HF), the terminal stage of various cardiovascular diseases, is marked by abnormal intercellular electrical signaling in cardiomyocytes (50). Recent studies have confirmed that Cx43 expression and functional abnormalities are closely linked to the occurrence and progression of HF. In end-stage HF patients and animal models, Cx43 expression is significantly downregulated, particularly in ventricular myocardial tissue (43). Moreover, Cx43 distribution is altered, with redistribution from intercalated discs to the lateral sides of cardiomyocytes (51).

In HF-related molecular mechanisms, oxidative stress and inflammatory signals are key regulators of Cx43 changes (47). Oxidative stress can lead to protein degradation and abnormal phosphorylation of Cx43, with increased dephosphorylation causing gap junction disassembly. This not only affects intercellular electrical conduction but also impacts mitochondrial function, promoting cardiomyocyte apoptosis (48). Inflammatory factors can suppress Cx43 expression by influencing its gene transcription and translation (43). Studies have found that inhibiting oxidative stress or inflammatory signals (e.g., the NF-κB pathway) can indirectly improve Cx43 expression and function, thereby alleviating HF progression (45).

Given its key role in cardiomyocyte communication, Cx43 is a crucial therapeutic target for HF (45). Targeted regulation of Cx43 expression and phosphorylation state, stabilizing its distribution in the cell membrane, and enhancing mitochondrial Cx43 function may offer new strategies for improving HF.

## 11 Summary

This article reviews the close relationship between Cx43 and various cardiac diseases, emphasizing the dynamic changes in Cx43 levels, distribution, and phosphorylation status in the occurrence and progression of cardiac diseases. In most cardiovascular diseases, Cx43 typically exhibits reduced expression, lateral distribution, and increased dephosphorylation, leading to intercellular communication barriers and mitochondrial dysfunction, thereby exacerbating myocardial injury. The relationship between Cx43 abnormal expression and various types of heart disease is summarized below (Table 4).

**Table 4:** Roles of Cx43 in cardiovascular diseases.

Disease type	Role of Cx43
Atrial Fibrillation	Abnormal distribution of Cx43
Cardiac Fibrosis	Cx43 dephosphorylation
Myocardial Infarction	Phosphorylation modification of Cx43 (serine 368 site)
Myocardial Ischemia-Reperfusion Injury	Cx43 dephosphorylation at the S282 site
Heart Failure	Downregulation and abnormal distribution of Cx43
Hypertrophic Cardiomyopathy	Downregulation of Cx43 gene and protein expression
Sudden Cardiac Death	Reduced expression and abnormal distribution of Cx43
Diabetic Cardiomyopathy	Loss of Cx43, lateral distribution, and excessive activation of hemichannels

In-depth research on Cx43 provides a new theoretical basis for understanding the pathogenesis, progression, and formulation of treatment strategies for cardiac diseases. However, the specific mechanisms still require further research and exploration.
